# Optimization and mechanisms of rapid adsorptive removal of chromium (VI) from wastewater using industrial waste derived nanoparticles

**DOI:** 10.1038/s41598-022-18494-0

**Published:** 2022-08-19

**Authors:** Hala M. Hamadeen, Elsayed A. Elkhatib, Mohamed L. Moharem

**Affiliations:** 1 Department of Soil and Water Sciences, Faculty of Agriculture (El-Shatby), Alexandria University, Alexandria, 21545 Egypt; 2 Regional Center for Food and Feed, Agricultural Research Center, Alexandria, Egypt

**Keywords:** Pollution remediation, Environmental chemistry

## Abstract

Nowadays, the existence of metal ions in the environment like chromium (VI) is of significant worry because of its high toxicity to many life forms. Therefore, in this study, an inexpensive and eco-friendly nano-adsorbent was produced from the waste of drinking water industry for effective elimination of Cr (VI) from wastewater. The mineralogical and morphological characterization and compositions of the bulk and nano- adsorbents were performed. The adsorption capabilities of nWTRs for Cr(VI) under different experimental conditions of adsorbent dosage, time, Cr (VI) concentration, solution pH, and competitive ions were investigated. The nWTRs adsorbent exhibits very rapid adsorption potential (92%) for Cr (VI) within the first 15 min. Langmuir model showed high predictive capability for describing Cr (VI) sorption equilibrium data. The estimated maximum sorption capacity (q_max_) of nWTRs and bWTRs was found to be 40.65 mg g^−1^ and 2.78 mg g^−1^ respectively. The sorption kinetics data of Cr (VI) were perfectly fitted to the model of second-order kinetics. High immobilization capability of nWTRs for sorbed Cr (VI) is evident as most of adsorbed Cr (VI) was associated with the residual fraction. The nWTRs efficiency of Cr (VI) removal from wastewater using batch and column techniques were 98.12 and 96.86% respectively. Electrostatic interactions, outer sphere complexation and pore filling are the main mechanisms suggested for binding of Cr(VI) with functional groups of nWTRs. This study demonstrates that the green low-cost nWTRs have the potential to decontaminate industrial wastewater effluents containing Cr (VI).

## Introduction

There is a global concern about the discharge of heavy metals into aquatic ecosystems^1^, because these toxic elements can lead to serious negative effects on ecological functions and human health^2^. Among the most toxic elements are chromium and its compounds. Chromium compounds are present in the effluents of many industries like tanneries, metallurgical, electroplating, textiles, and chemical industries^3,4^. Chromium ions present in the aquatic environment in chromium III (Trivalent) and chromium VI (Hexavalent) forms. The Cr(III) is an important nutrient in humans^5^, whereas hexavalent is 100 times more toxic than trivalent due to its high oxidative potential and ease of penetration via biological membranes^6^.

The global market for leather is booming, and the industry has a huge negative environmental impact because of the utilization of toxic chromium used in the tanning process, and thus it is considered one of the biggest pollutants worldwide. Approximately 85% of all leathers produced are tanned with chromium-based process technology, due to its low costs, fast processing, color stability and a high degree of thermal resistance^7^.The tanning process uses chromium salt with collagen components to prevent water from penetrating the pores of the skin and causing rot. In the chromium tanning process, the skin consumes 60 to 80% of chromium applied and the remainder is frequently released into the drainage system, which has major environmental consequences^8^.

Many techniques are used for chromium (VI) removal from different wastewaters namely adsorption^9,10^, nanotechnology treatments^11,12^, membrane filtration^13^, ion exchange, chemical precipitation, electrochemical, and advanced oxidation processes^14^. However, the disadvantage of these techniques such as time-consuming, laborious, costs, generation of sludge that causes disposal problems, and the production of a secondary pollutant are limiting their applicability in a real situation^15^. Therefore, developing of green, inexpensive and efficient methods for elimination of metals and other pollutants, while avoiding adverse effects on treated systems are needed.

Water treatment residual (WTRs) is a generated waste product of drinking water purification industry when Al_2_ (SO_4_)_3_ is used during the coagulation process. Millions of tons of WTRs are generated every year globally which signify an environmental-economical challenging problem. Amorphous Al (OH)_3_ and SiO_2_ mainly constitute WTRs with small percentages of other oxides. The application of WTRs as efficient and low-cost adsorbent for the remediation of heavy metals contaminated soil and water have been reported as a potential and attractive remediation route^16,17^. Because WTRs is not listed as a harmless “waste” in EU regulations^18,19^, the use of WTRs as a green adsorbent would greatly strength the sustainable remediation and environmental conservation.

Application of nanosized materials in wastewater treatment has gained much attention lately and has resulted in enhanced performance over their bulk counterparts due to their unique physicochemical characteristics such as small sizes (< 100 nm) and high adsorption capacities^20–24^. Thus, substituting the expensive commercial nanomaterials by low-cost nanostructured adsorbents with competent adsorption performance, derived from industrial waste product exhibits a new concept and encouraging substitute due to its sustainability, abundant, economic, and environmental footprint^25^. To the best of authors' knowledge, information is very limited on the production of nanostructured sorbent from water industry byproducts for use in removal of Cr (VI) contaminant from industrial effluents. Therefore, the objectives of the present research were to: (1) transform the byproducts of drinking water industry (WTRs) to valuable nanostructured WTRs (nWTRs) for enhanced removal of Cr (VI) from wastewater effluents, (2) determine the operating status of Cr (VI) sorption onto nWTRs through adsorption isotherms and kinetic studies and (3) examine Cr (VI) adsorption mechanisms onto nWTRs.

## Materials and methods

### Production of nanostructured WTRs (nWTRs)

The WTRs waste was collected from the drinking water treatment plant (Alexandria, Egypt), air dried for a period of 3 weeks at room temperature, then ground and sieved using 2 mm sieve. The obtained product (bulk WTRs; bWTRs) was stored for analyses. Samples of bWTRs were sieved using 51 μm sieve and were ground using Fritsch planetary mono ball mill (Fritsch, Germany) to obtain WTRs nanoparticles (nWTRs) with particles size < 100 nm following the method of Elkhatib et al.^26^.

### Characterization

Scanning electron microscopy (SEM) with energy dispersive X-ray (EDX), transmission electron microscopy (TEM) (INCAx-Sight model 6587, Oxford Instruments, UK)^27^, and Fourier transform infrared spectroscopy (FTIR) were used to assay the properties, element compositions, and functional groups of nWTRs before and after loading with Cr (VI).

### Sorption kinetics

Sorption kinetic studies were conducted in 50 ml centrifuge tubes containing 100 mg/10 ml of nWTRs and Cr(VI) with 350 mgl^−1^ initial concentration. The mixtures were shaken (end-over-end shaker) for various time intervals (5, 30, 60, 240,480, and 1440 min) at three different pH levels (5, 7 and 9). Solution pH was adjusted with hydrochloric acid (HCl) and sodium hydroxide (NaOH) with concentrations of 0.1 mol l^−1^. The liquors were centrifuged and filtered (0.45 um Millipore filter). Using inductively coupled plasma spectrometry (ICPS), the supernatant was analyzed for Cr(VI), and the obtained data were fitted to five kinetic models.

### Sorption isotherms

The Cr(VI) sorption equilibrium studies were determined using two different particle sizes of WTRs (2 mm and less than 100 nm) at different concentrations (0, 5 20, 40.80 and 160 mgl^−1^) in a background electrolyte of 0.005 M KNO_3_. Solutions obtained were placed in contact with WTRs materials in 50 ml capped polyethylene tubes. The bulk WTRs—Cr(VI) mixtures were equilibrated on a slowly rotating rack for 1440 min at normal pH (7.2), and then removed for centrifugation (4000 rpm) for 15 min, and the data obtained were fitted into different isotherms models. The solid nWTRs—Cr(VI) mixtures after sorption experiments were recovered and examined for sorbed Cr(VI) utilizing SEM, TEM and FTIR analysis.

To study the impact of nWTRs dosage on the Cr(VI) removal, 20, 50, or 100 mg of nWTRs sample were added to 10 ml portions of Cr (VI) with concentration of 200 mgl^−1^. Competitive adsorption tests in batch (single and multi- ions systems) were performed using nWTRs and solutions containing equal amounts of As (V), Hg (II), and Cr (VI) (multi-ion system) with different concentrations (0, 5, 20, 40.80 and 160 mgl^−1^) in background electrolyte (0.005 M KNO_3_). The suspensions were equilibrated for 1440 min.

## Results and discussion

### Characteristics and chemical composition of nWTRs

**Figure 1:**
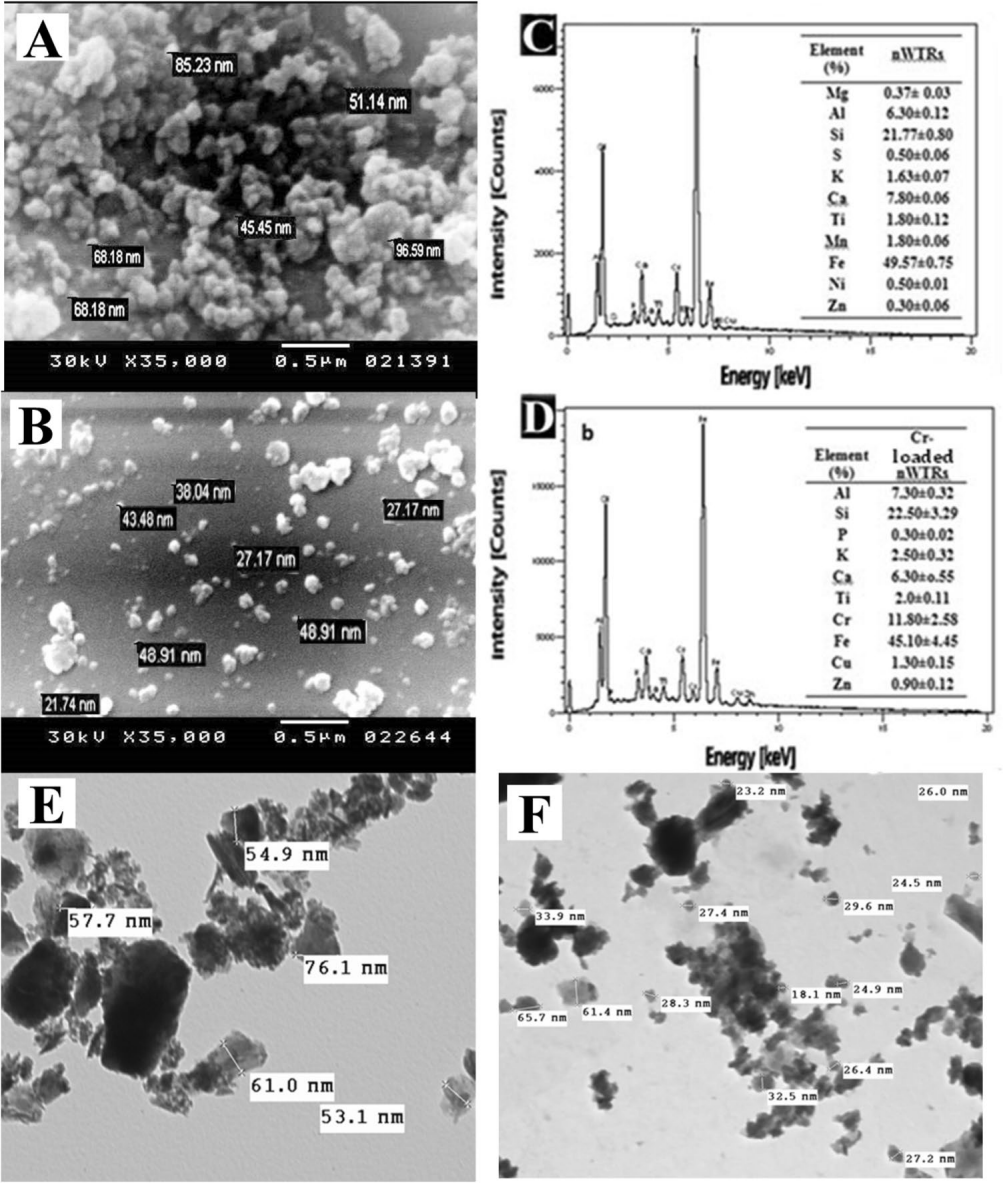
Scanning electron microscopy (SEM) image and energy dispersive X-ray (EDX) elemental distribution of nWTRs (**A**–**C**), the Cr(VI)—loaded nWTRs (**B**–**D**), Transmission electron microscopy (TEM) image of nWTRs (**E**) and Cr(VI)—loaded nWTRs (**F**).

SEM and EDX are powerful tools for examining the structure of surface morphology and the elemental features of the sorbents^28^. The particles morphology and elemental contents of nWTRs and Cr(VI) loaded nWTRs are shown in Fig. 1. The SEM image of the nWTRs sample (Fig. 1A) revealed that the nanoparticles were spherical, with particle sizes < 100 nm in diameter. EDX elemental analysis (Fig. 1C) exhibited the major elements of nWTRs (iron, silicon, calcium, and aluminum) with small amounts of potassium, sulfur, titanium, and manganese. The SEM image of Cr(VI) loaded nWTRs (Fig. 1B) revealed a coating layer of Cr(VI) adsorbed on the nWTRs surface, indicating that the reaction occurred on the nWTRs surface. According to the EDX analysis (Fig. 1D), a Cr (VI) peak (11.80%) is noticed among the elements detected in Cr(VI)-loaded nWTRs.

The TEM image of the nWTRs and Cr(VI) loaded nWTRs (Fig. 1E,F) revealed that the nWTRs are somewhat agglomerated with particle sizes ranging from 18.1 to 76.1 nm.

BET specific surface area (SSA) analysis of bulk and nano particles of WTRs are 53.1 and 129 m^2^g^−1^, respectively. It is clear that SSA of nWTRs is 2–3 times higher than that of bWTRs which makes it ideal candidate for use in water treatment.

### Surface functional groups of nanoscale WTRs

**Figure 2:**
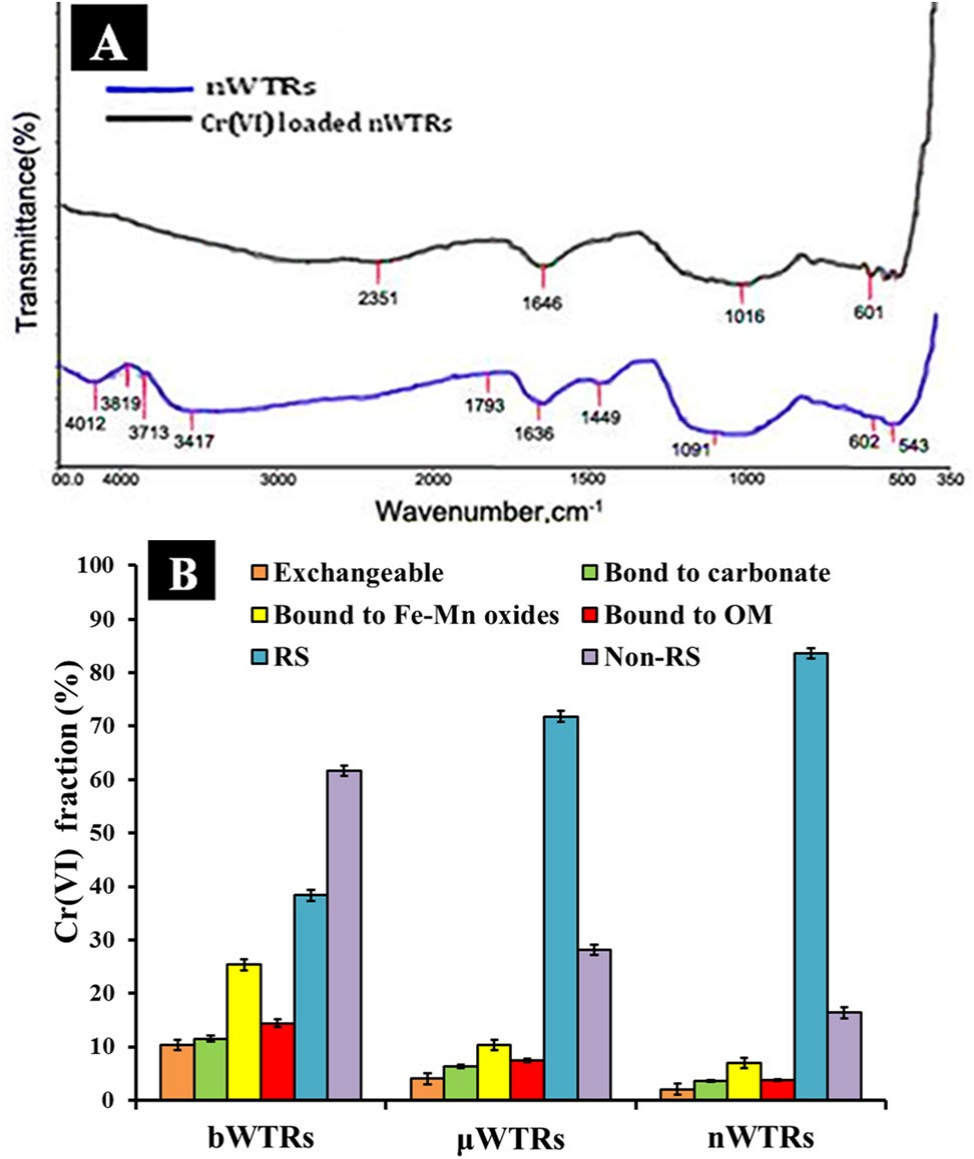
FTIR spectrum analysis of nWTRs before and after Cr(VI) adsorption (**A**), and Fractionation of adsorbed Cr (VI) on different particle sizes of WTRs Notice that Cr (VI) adsorbed on nWTRs was mostly associated with the less mobile fraction (residual fraction, RS), which indicates the high capability of nWTRs to immobilize Cr (VI) (**B**).

The sorbents characterization by Fourier transmission infrared (FTIR) spectra reveals the functional group in the sorbent's structure^29^ which is crucial for understanding mechanisms of metals removal by the solid surfaces. FTIR spectroscopy results of nWTRs and Cr(VI) loaded nWTRs are presented in Fig. 2A. The bands detected at 3619 cm^−1^, 3417 cm^−1^ and 1636 cm^−1^ in the FTIR spectrum of nWTRs (Fig. 2A lower) are assigned to dangling O–H bonds, the HO–H stretching and bending vibrations respectively^22,30^. Additionally the observed bands at 1449 cm^−1^,1091 cm^−1^, and 601 cm^−1^ are assigned to Fe OH bending vibration-modes of feroxyhyte^30,31^ and bending vibration of Al single bond OH group^32,33^. After Cr(VI) adsorption, the bands and peaks between 3417 and 4012 cm^−1^ (referred to the dangling O–H bonds and O–H bending vibration) as well as the peak at 1793 cm^−1^ completely disappeared. In addition, shifting of the peak at 1626 cm^−1^ to higher wavenumber (1646 cm^−1^) and shifting of the peak at 1091 cm^−1^ referred to O–Al–O bending vibration was shifted to lower wave number(1016 cm^−1^) were observed. Disappearance and shifting of peaks and bands suggest molecular interaction and adsorption of Cr(VI) on nWTRs through functional groups^34^. The FTIR results apparently indicate that presence of O–H and O–Al–O functional groups play significant role in Cr(VI) adsorption on nWTRs adsorbent.

### Fractionation of the Cr(VI)-loaded nWTRs and Cr(VI) mobility

To assess the potential Cr(VI) mobility sorbed onto nWTRs compared to that sorbed onto μWTRs and bWTRs, the distribution of Cr(VI) in the fractions of the sorbents studied was performed using the widely employed sequential extractions(fractionation) scheme^35,36^. According to this scheme, the unstable Cr (VI) in the non-residual fraction is more mobile than the Cr(VI) in the residual (RS) fraction. Thus, correlating Cr(VI) data with sorbent fractions would determine the mobility of Cr(VI) bound to various sorbent fractions. Figure 2B. Clearly exhibits that Cr(VI) adsorbed by bWTRs was mostly correlated with the more mobile Non-RS fraction (61.67%) while 83.64% of Cr(VI) on nWTRs was related to the less mobile fraction (RS). These variations are referred to the larger surface area and higher adsorption capacity of nWTRs.

### Adsorption equilibrium study

Adsorption isotherm models are extremely useful in determining adsorption capacity of the sorbents and in selecting the most suitable sorbent under optimal experimental conditions^37,38^. The experimental adsorption equilibrium data of the present study were analyzed using six adsorption isotherm models (Table 1). The determination coefficient (R^2^) and the standard error of estimate (SE) values presented in (Table 1) are indicators of how well the model fits the data. The highest R^2^ value and the lowest SE value of Langmuir model (Table 1) signalize the best fit of the model to Cr(VI) adsorption data. The Lowest SE values of 0.00062 and 0.000014 (Table 1 and Fig. 4A) of Cr(VI) adsorption onto bWTRs and nWTRs sorbents respectively affirmed that Cr(VI) adsorption onto the studied sorbents fitted best to Langmuir model. The calculated maximum sorption capacity (q_max_) of nWTRs and bWTRs were found to be 40.65 mg and 2.78 mg Cr (VI) g^−1^ respectively. The calculated q_max_ value of nWTRs was 15 times higher than that of bWTRs.

**Table 1:** The parameters of isotherms models for Cr(VI) adsorption onto the two particle sizes of WTRs.

Models	Description	Parameter	WTRs	
Adsorption isotherms			bWTRs	nWTRs
Freundlich $q_{e} = K_{F} C_{e}^{1/n}$	*K*_*F*_ = constants of Freundlich (the adsorption volume of the adsorbent)	K_F_ (mL/g)	56.92	154,199.1
		1/n	0.838	0.77
	1/n = constants (intensity of the analytes' sorption)	R^2^	0.95	0.99
		SE	0.341	0.151
Langmuir $q_{e} = \frac{{q_{{max K_{L} C_{e} }} }}{{1 + K_{L} C_{e} }}$	*q*max = maximum adsorption capacity	q_max_ (mg/g)	2. 78	40.65
	K_L_ = constant of Langmuir ( free energy of adsorption )	K_L_ (L/mg)	0.0186	14.7
		R^2^	0.99	0.99
		SE	6.22E-04	1.41E-05
Temkin $\theta = \frac{RT}{{\Delta Q}}lnK_{0} C_{e}$	Δ*Q* = variation of adsorption energy (− Δ*H*)	∆Q (KJ/Mol)	14.55	15.09
	*K*_0_ = constant of Temkin	K_0_ (L/mg)	0.448	347.48
	*T* = temperature (K)	R^2^	0.65	0.86
	*R* = universal gas constant	SE	0.214	0.133
Fowler–Guggenheim(FG) $K_{FG} C_{e} = \frac{\theta }{1 - \theta }exp\left( {\frac{2\theta w}{{RT}}} \right)$	= interaction energy between adsorbed molecules	W (kJ/mol)	2.419	0.834
		K_FG_ (L/mg)	0.0125	12.71
	*K*_FG_ = constant of Fowler–Guggenheim	R^2^	0.63	0.55
	θ = fractional coverage	SE	0.543	0.213
Kiselev ${\text{k}}_{1} {\text{C}}_{{\text{e}}} = { }\frac{\theta }{{\left( {1 - { }\theta } \right)\left( {1 + {\text{k}}_{{\text{n}}} \theta } \right)}}$	K_1_ = constant of Kiselev	k_1_ (L/mg)	0.0178	14.361
	*kn* = constant (complex formation between adsorbed molecules )	kn	1.140	0.181
		R^2^	0.97	0.99
		SE	0.0533	11.85
Hill–deBoer $K_{1} C_{e} = \frac{\theta }{1 - \theta }exp\left( {\frac{\theta }{1 - \theta } - \frac{{K_{2} \theta }}{RT}} \right)$	*K*_1_ = constant of Hill-de Boer	K_1_ (L/mg)	0.00659	7.60
	*K*_2_ = constant (interaction between adsorbed molecules )	K_2_ (kJ/mol)	19.154	13.63
		R^2^	0.87	0.87
		SE	1.109	0.761

### Effect of the varying different parameters on Cr (VI) adsorption. Initial Cr (VI) concentration

**Figure 3:**
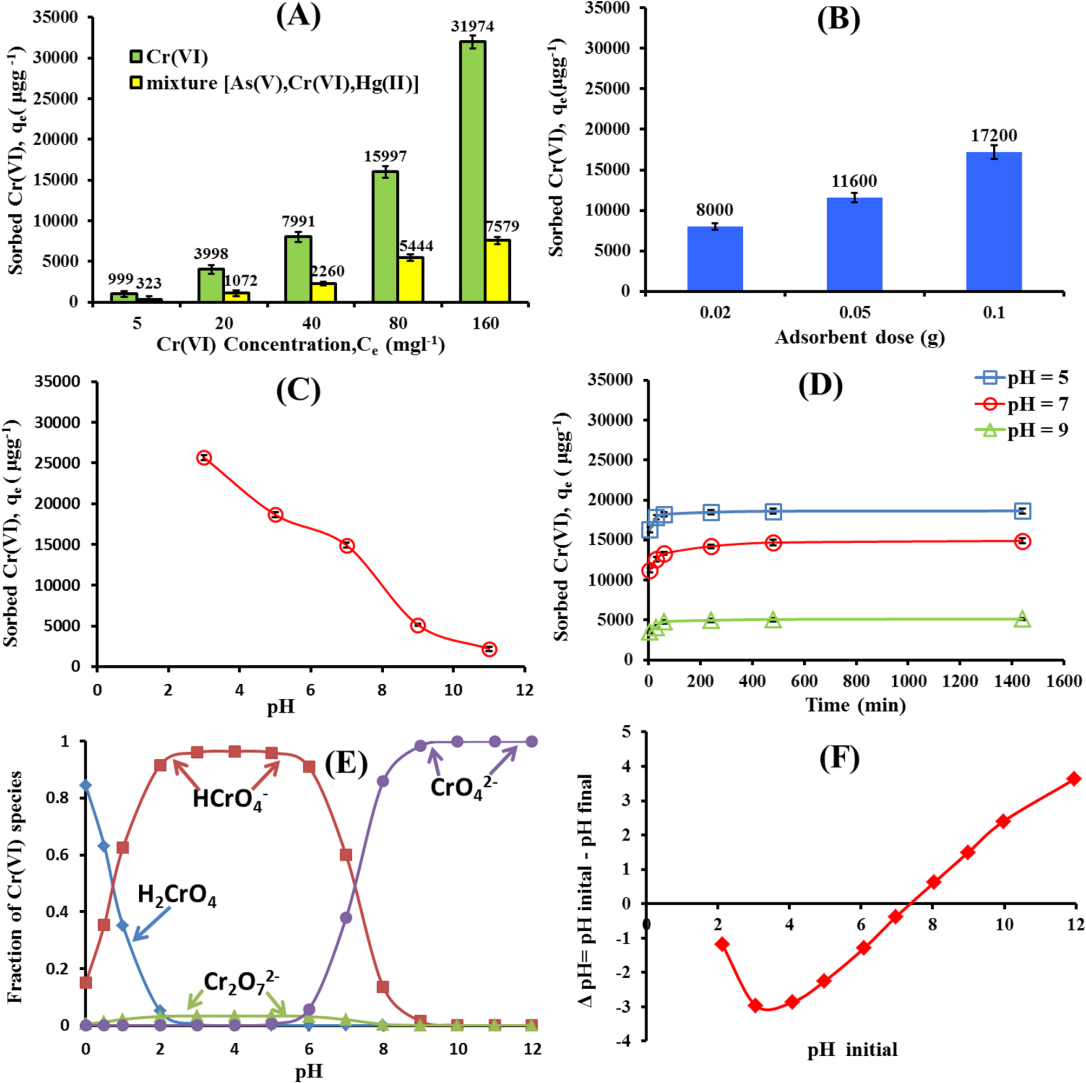
Effect of initial Cr(VI) concentration and competing ions (**A**), adsorbent dose (**B**) solution pH (**C**) and Contact Time (**D**) on the Cr(VI) adsorption by nWTRs. Cr(VI) species (**E**), and zero point charage of nWTRs (**F**).

The influence of initial Cr (VI) concentration at a range of 5 to160 mgl^−1^ on adsorption capacity of nWTRs is shown in Fig. 3A. The results show that increasing Cr (VI) initial concentration has led to increasing **Cr (VI)** adsorption onto nWTRs adsorbent. The Cr (VI) adsorbed by nWTRs increased from 999 to 31,974 μgg^−1^ with increasing Cr (VI) initial concentration from 5 to 160 mgl^−1^ due to the increased mass transfer driving force which encouraged the adsorbate transport from the solution to the adsorbent surface^39^ and enhanced the interaction between adsorbent and adsorbate^40^.

### Competitive ions

The competition effects of the amounts of Cr (VI) adsorbed by nWTRs in presence or absence of competing cations such as As and Hg at concentrations equal to Cr (VI) concentration were investigated in single and multi-element systems (Fig. 3A). As shown, the power of nWTRs for removing Cr (VI) was highly affected as a result of As and Hg presence in the solution. For instance, at high Cr (VI) initial concentration, Cr (VI) removal dramatically decreased from 99 to 24% due to competition between Cr and As/Hg cations for available sorption sites of nWTRs (Fig. 3A). Similarly, Jain et al.^41^ indicated that the adsorption capacity of sunflower plant biomass-based carbons for Cr (VI) was reduced in binary and tertiary multi-element systems because of the progressive interference and competition of cations for binding sites on the adsorbent.

### Adsorbent dosage

The effect of nWTRs dose (20–100 mg) on Cr(VI) adsorption was studied. Increasing nWTRs dosage from 20 to 100 mg increased the adsorption efficiency of the adsorbent and the adsorbed amount of Cr(VI) increased from 8000 to 17,200 μgg^−1^ (Fig. 3B). This is because of the greater surface area and the availability of larger number of adsorption sites for HCrO_4_^−^ and Cr_2_O_4_^−^ ions^42^. Therefore, the dose of 100 mg (nWTRs) was used in the optimization studies.

### Solution pH

The Cr (VI) in solution is mostly found in the forms of H_2_CrO_4_, HCrO_4_^−^, Cr_2_O_7_^2−^, CrO_4_^2−^ depending on Cr(VI initial concentration and pH as shown in Fig. 3E. The pH value of sorbent is a significant parameter for metal adsorption^43–45^. Thus, the impact of pH solution on Cr(VI) removal by nWTRs in the pH range (3–11) was examined. Figure (3C) clearly shows that Cr(VI) adsorption on to nWTRs is reduced significantly with increasing pH until it reaches pH 11. For better understanding of Cr(VI) adsorption mechanisms, the point zero charge (pHzpc) of nWTRs was determined and found to be 7.43 (Fig. 3F). At pH values lower than 7.43 the nWTRs surface is positively charged whereas at higher pH values than7.43, the nWTRs surface is negatively charged^46,47^. Because in acid solutions Cr (VI) exists mainly as negatively charged anions (HCrO_4_^–^–Cr_2_O_7_^2–^), and nWTRs surface is positively charged, strong electrical interactions between Cr(VI) species and positively charged sites on nWTRs surface are taking place and as a result the adsorption capacity of nWTRs increased (Fig. 3C). Conversely, as the pH of the nWTRs increased above its zero point charge (pH > pHzpc), the number of sites that carried negative charges increased while the number of sites that carried positive charges decreased, resulting in a decrease in the adsorption capacity of nWTRs for Cr (CrO_4_^2−^ is the main ionic species)^48,49^.The obtained result are in agreement with the previous work reported using various adsorbents for Cr(VI) removal^43,50–52^.

**At acidic condition:**

$${\mathbf{HCr}}_{2} {\mathbf{O}}_{7} \to {\mathbf{H}}^{ + } + {\mathbf{Cr}}_{2} {\varvec{O}}_{7}^{2 - }$$

$${\mathbf{H}}_{2} {\varvec{Cr}}{\mathbf{O}}_{4} \to {\mathbf{H}}^{ + } + {\mathbf{HCrO}}_{4}^{ - }$$

**At basic condition:**

$${\mathbf{Cr}}_{2} {\mathbf{O}}_{7}^{2 - } + {\mathbf{OH}}^{ - } \to {\mathbf{HCrO}}_{4}^{ - } + {\mathbf{CrO}}_{4}^{2 - }$$

$${\mathbf{HCrO}}_{4}^{ - } + {\mathbf{OH}}^{ - } \to {\mathbf{CrO}}_{4}^{2 - } + {\varvec{H}}_{2} {\varvec{O}}$$

### Contact time

The effect of contact time (5 to 1440 min) on Cr (VI) adsorption at pH values (5, 7and 9) was studied. As appeared in Fig. 3D, the Cr(VI) adsorption by nanostructured WTRs was instantaneous. Approximately 92% of Cr(VI) was adsorbed within the first 15 min onto nWTRs adsorbent and reached equilibrium in almost one hour with Cr(VI) adsorption efficiency of ~ 99%. The reduced removal efficiency of nWTRs for Cr(VI) at higher pH values(7 and 9) is evident (Fig. 3D) due to the positively charged nWTRs surfaces under acidic conditions.

### Modelling adsorption kinetics

**Figure 4:**
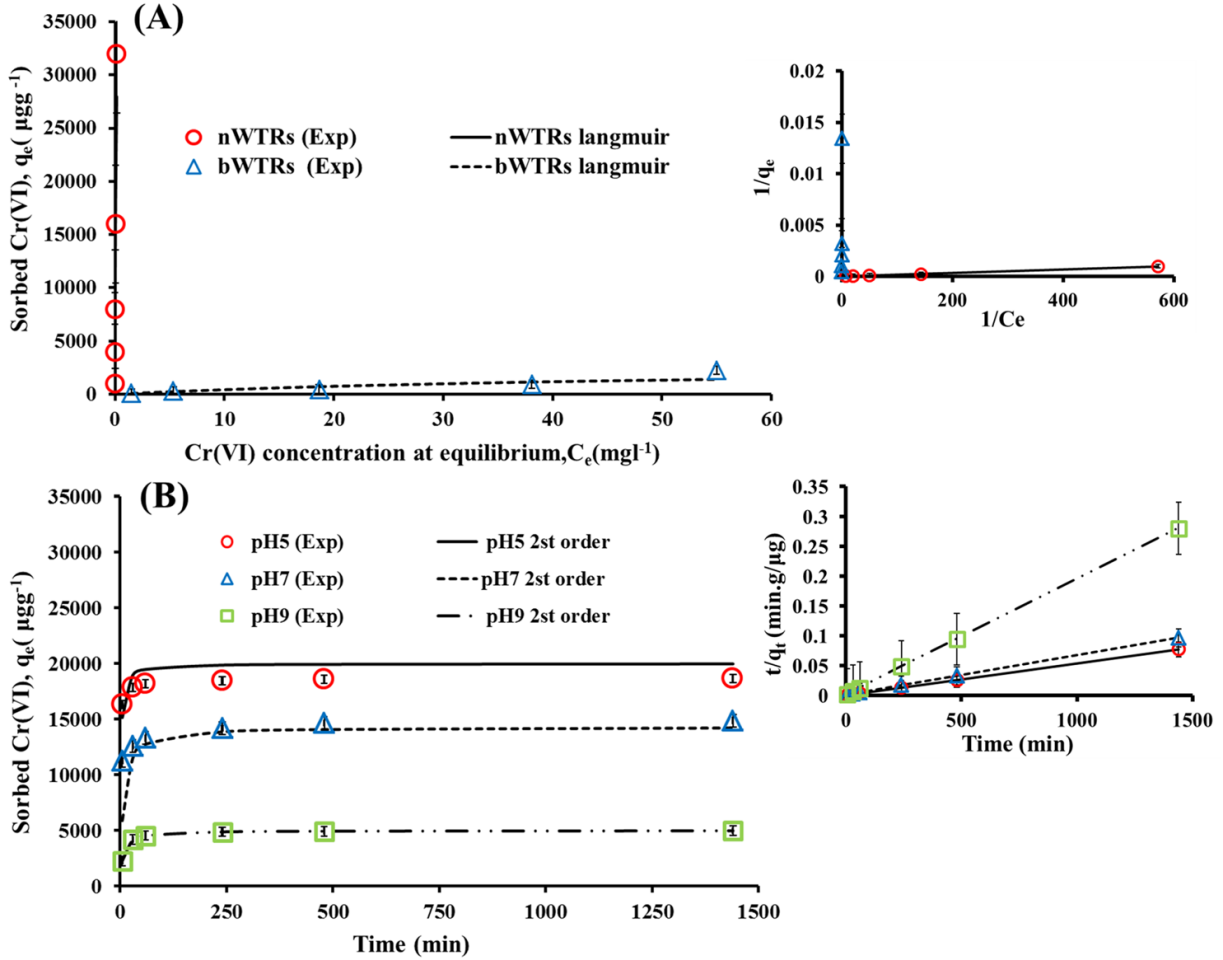
The well fitted adsorption models (**A**) Langmuir and (**B**) Second -order for the Cr(VI) adsorption. The side shows the linear forms of the models (The error bars represent the standard error of mean).

Five models were utilized to model the Cr(VI) adsorption kinetics data by nanoscale WTRs at three pH values (5, 7, and 9)^53^. The adsorption kinetics models used were evaluated to determine the best fit model that could reproduce the results. Table 2 shows the kinetic models tested and its parameters. Generally, higher R^2^ values and lower SE values better describe the kinetic model of adsorption process. It can be clearly seen (Table 2) that kinetics data of Cr(VI) adsorption by nanoscale WTRs at pH values (5, 7, and 9) are best described by the second-order model based on the lowest standard errors (SE) values and the highest determination coefficient (R^2^) of the second-order model (Table 2 and Fig. 4B).

**Table 2:** The parameters of kinetic models for Cr(VI) adsorption by the three various pH values.

Models adsorption kinetics	Description	Parameter	pH5	pH7	pH9
First order $$q_{{\text{t}}} = q_{{\text{e}}} \left( {\begin{array}{*{20}c} {1 - } & {e^{{ - K_{a} t}} } \\ \end{array} } \right)$$	q or q_t_ = Amount of Cr(VI) adsorbed at time t	K_a_ (min^−1^)	0.0076	0.0055	0.0064
	q_e_ = Amount of Cr(VI) adsorbed at equilibrium	q_e_ (mg/g)	1174.94	2795.31	994.36
	Ka = Apparent adsorbed rate coefficient	R^2^	0.91	0.97	0.89
		SE	0.546	0.226	0.513
Second order $q_{t} = \frac{{k_{b} q_{e}^{2} t}}{{1 + k_{b} q_{e} t}}$	K_b_ = Apparent adsorbed rate coefficient	K_b_ (min^−1^)	3.13E−05	9.80E−06	3.33E−05
		q_e_ (mg/g)	20,000	14,285.7	5000
		R^2^	1	1	1
		SE	3.20E−05	2.75E−04	5.21E−04
Elovich $q_{{\text{t}}} = \frac{1}{{\upbeta }}Ln\left( {1 + \alpha \beta t} \right)$	β = Constant related to the extent of surface coverage	α (mg/g min)	3.73E + 20	3.48E+09	2.18E +07
		β (mg/g)	0.0026	0.0015	0.0035
	α = The initial adsorbed rate	R^2^	0.81	0.97	0.89
		SE	426.89	249.67	233.97
Parabolic diffusion $q = k_{{\text{d}}} t^{1/2}$	K_d_ = Apparent diffusion rate coefficient	K_d_ (mg/g min^1/2^)	46.055	89.629	36.648
		R^2^	0.47	0.73	0.60
		SE	719.15	804.64	446.60
Power function $q = k_{{\text{a}}} C_{{\text{o}}} t^{{1/{\text{m}}}}$	Ka = Apparent adsorbed rate coefficient	K_a_ (min^−1^)	16,210.63	10,570.61	3347.34
		1/m	0.0223	0.0511	0.0664
	1/m = Constant	R^2^	0.80	0.96	0.88
	Co = Initial Cr(VI) concentration	SE	0.01096	0.00976	0.02495

### Proposed mechanisms of Cr (VI) removal using nWTRs sorbent

**Figure 5:**
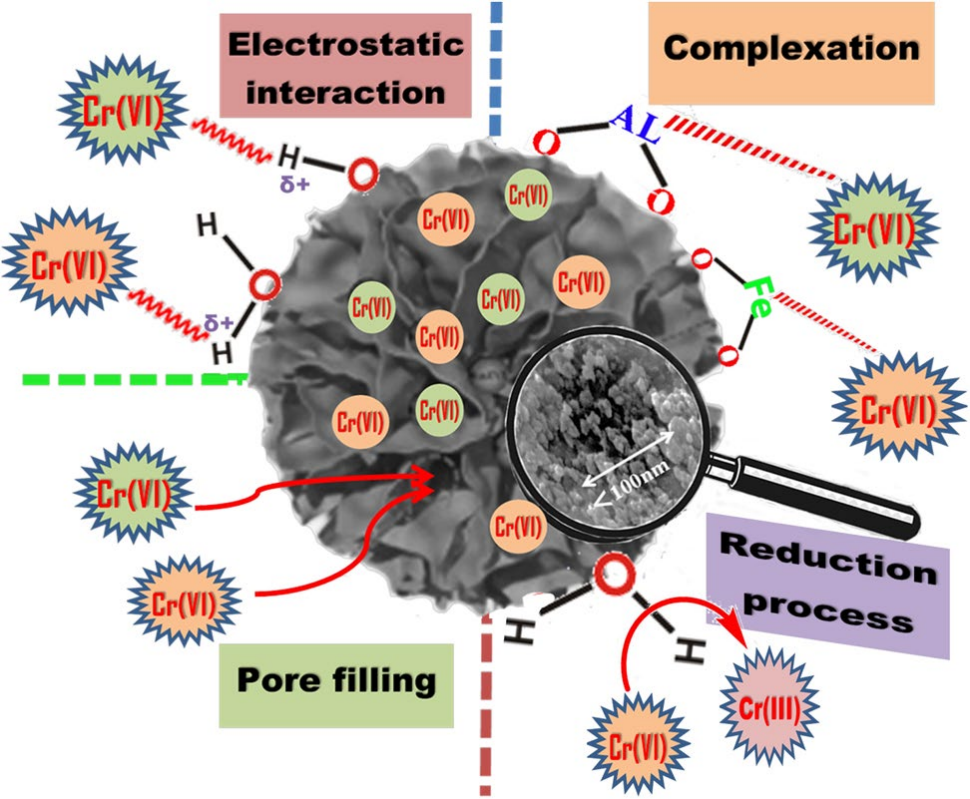
Schematic representation of Cr(VI) removal mechanism by nWTRs.

The results of Cr(VI) adsorption studies and FTIR analysis of nWTRs and Cr-loaded nWTRs revealed that O–H, O–Al–O and Fe hydroxide functional groups are the main functional groups that significantly facilitate Cr(VI) reactivity with nWTRs. Consequently, the likely mechanisms of Cr(VI) adsorption onto nWTRs surface are suggested in Fig. 5. The proposed figure exhibits the different interactions involved in the Cr(VI) adsorption process including electrostatic interactions (i), Complexation (ii) Pore filling (iii) and Reduction process (iv):

(i) *Electrostatic interaction* The negatively charged Cr(VI) species, under acidic condition, were favorably migrated to the positively charged surfaces of nWTRs (Al, Fe oxides and OH atoms) (The OH atoms is protonated under acidic conditions to produce—OH_2_^+^)^54^.
(ii) *Complexation* The Cr(VI) ions could form inner sphere complexes with Fe/Al oxides of nWTRs with a more pronounced effect in acid conditions^55^.
(iii) *Pore filling* Chromium ions possibly trapped in the micropores of nWTRs surface because the pores size of the nWTRs are larger than the radiuses of HCrO_4_^−^ and Cr_2_O_7_ ions^56^.
(iv) *Reduction process* At a lower pH, Cr(VI) may be reduced to Cr(III)^57^, which could then be chelated with Al/Fe oxides on the surface of nWTRs.

### The feasibility of nWTRs application for Cr(VI) removal from wastewater

The ability of nWTRs to efficiently remove Cr(VI) from actual wastewater was evaluated using batch and packed-bed column techniques. Samples from wastewater discharged from the food processing plant were collected in polyethylene bottles. The pH, electric conductivity (EC), Cr(VI) concentration, major and minor elements, of the wastewater samples were all measured and are presented in Table S1. All wastewater samples (one Liter) were spiked with 100 mgl^−1^ Cr(VI) solution. A 100 mg nWTRs was added to each wastewater sample. The mixture was shaken for 120 min, then centrifuged and the supernatant solutions were filtered and analyzed.

### Packed-bed column technique

**Figure 6:**
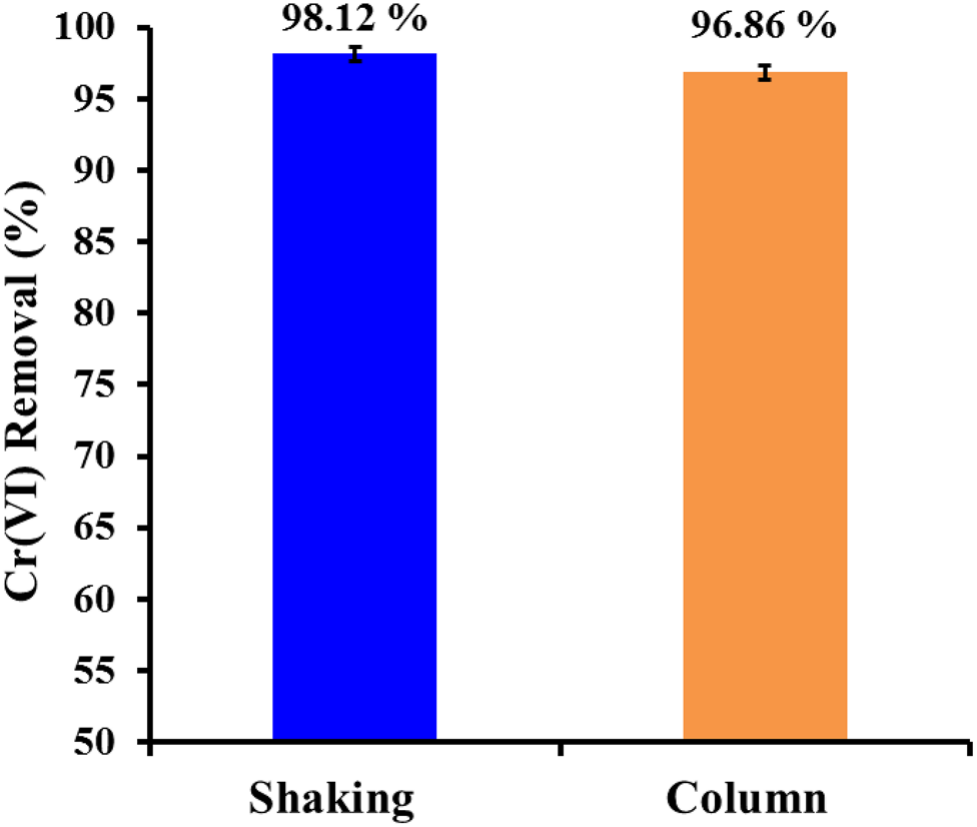
Efficiency of the nWTRs for Cr(VI) removal from wastewater.

The columns were made of plastic (UV stabilized PVC) with 25 cm length and internal diameter of 1.2 cm. Each column was filled with 600 mg nano- WTRs and sand at a specific ratio (5 g fine sand and 5 g coarse sand). The structure of columns bottom consist of a wool layer and a filter paper to preserve the solid contents of the column. Wastewater effluents spiked with Cr(VI) (100 mgl^−1^) were infiltrated across the column downward at low flow rate of 2 ml/min. The leachate was obtained at regular time intervals and analyzed. The efficiency of nWTRs using a real chromium contaminated wastewater sample, in batch (shaking) and packed-bed column were 98.12 and 96.86 respectively (Fig. 6).

### Comparison of adsorption capacity of nWTRs and other adsorbents for Cr(VI) removal

The maximum adsorption capacity (qmax) of nWTRs for chromium is compared with different adsorbents (Table 3). It can be seen that the nanostructured WTRs outperformed all the listed adsorbents in Cr(VI) removal from aqueous solutions.

**Table 3:** Maximum adsorption capacities (qm) of Cr(VI) adsorption on nWTRs and the other adsorbents documented in the literature.

Adsorbents	Adsorption capacity (qmax) (mg/g)	References
Nano Water Treatment Residuals (nWTRs)	40.65	Present study
Bulk Water Treatment Residuals (bWTRs)	2.78	Present study
Humus-supported nanoscale zero-valent iron	40.40	^58^
Fe modified activated carbon	34	^59^
NH2-amorphous silica nanoparticle	34	^60^
AC/bentonite/magnetite nanocomposite	29.32	^61^
Bagasse magnetic biochar (BMBC)	29.08	^62^
MgO/Fe3O4nanocomposite	23.9	^63^
Bentonite supported nZVI	22.67	^64^
Maghemite nanoparticles	19.20	^65^
Mesoporous magnetic γ-Fe2O3	16	^66^
Multi-wall carbon nanotubes	2.48	^67^
Goethite	6.62	^68^
Zeolite NaX	6.41	^69^
Activated alumina	1.6	^54^

## Conclusions

A low-cost nano-adsorbent (nWTRs) produced from byproducts of water industry has proven to be highly effective in removing Cr(VI) from contaminated wastewater. The linearized Langmuir model was statistically superior to the other models tested and showed excellent ability to model Cr(VI) adsorption data with good accuracy. The calculated maximum sorption capacity (qmax) of nWTRs (40.65 mgg^−1^) for Cr(VI) was 15 times higher than qmax of bulk WTRs (2.78 mgg^−1^). The second-order model best described Cr(VI) sorption kinetics data. FTIR and SEM–EDX spectra analysis suggests that the hydroxyls may act as the surface active sites for Cr(VI) binding to nWTRs surfaces. The efficiency of nWTRs in removing Cr (VI) from wastewater effluents using batch and column techniques were 98.12% and 96.86% respectively. It is, therefore, suggested the use of nWTRs as a powerful and rapid sorbent for elimination of Cr (VI) from industrial wastewater effluents.

## Supplementary Information

Supplementary Information 1.

Supplementary Information 2.

Supplementary Information 3.

Supplementary Information 4.

## Data Availability

All data generated or analyzed during this study are included in this published article and its supplementary information files.
