# Supplementary material for: Optimization and mechanisms of rapid adsorptive removal of chromium (VI) from wastewater using industrial waste derived nanoparticles
Source: Sci Rep. 2022 Aug 19;12:14174. doi: 10.1038/s41598-022-18494-0 (PMC9391442; doi:10.1038/s41598-022-18494-0)
Supplement: Supplementary file 4 — Supplementary Information 4. [file 41598_2022_18494_MOESM4_ESM.docx]

**Table S1.**  **Chemical characteristics of wastewater:**

| **Characteristics** | **Units** | **Value**† |
| --- | --- | --- |
| **pH** |  | **8.1** |
| **EC** | **dsm^-1^** | **3.11±0.11** |
| **Chloride** | **mgl^-1^** | **22.15±1.65** |
| **Nitrate** | **mgl^-1^** | **18.09±2.54** |
| **Phosphate** | **mgl^-1^** | **6.55±0.33** |
| **Sodium adsorption ratio(SAR)** |  | **6.12±0.89** |
| **Pb** | **mgl^-1^** | **2.22±0.05** |
| **As** | **mgl^-1^** | **3.00±0.09** |
| **Cr** | **mgl^-1^** | **0.40±0.03** |
| **Cu** | **mgl^-1^** | **0.09±0.01** |
| **Cd** | **mgl^-1^** | **0.04±0.002** |

†Means of three samples ± SD.
